# Mental health, perceived social support, and sense of belonging among immigrants

**DOI:** 10.1371/journal.pone.0343075

**Published:** 2026-02-24

**Authors:** Zahra Jafari, Mohammad Habibnezhad, Maryam Mirzaei Hotkani, Emily Balkam, Kolten C. MacDonell, Ellen Hickey

**Affiliations:** 1 School of Communication Sciences and Disorders (SCSD), Dalhousie University, Halifax, Nova Scotia, Canada; 2 Department of Psychology and Neuroscience, Dalhousie University, Halifax, Nova Scotia, Canada; 3 Department of Geriatric Medicine, Dalhousie University, Halifax, Nova Scotia, Canada; 4 Department of Otolaryngology-Head & Neck Surgery, Dalhousie University, Halifax, Nova Scotia, Canada; 5 Nova Scotia Health Authority, Department of Family Practice, Halifax, Nova Scotia, Canada; Access Alliance Multicultural Health and Community Services: Access Alliance, CANADA

## Abstract

**Objectives:**

Immigrants often encounter unique challenges that can adversely affect their mental health and social well-being. This research aimed to examine the mental health status (anxiety and depression), perceived social support, and sense of belonging among immigrants from non-English-speaking backgrounds in Canada, and to identify contributing factors.

**Methods:**

The research included 144 Farsi- and Arabic-speaking adults, with a mean age of 36.50 years; 51.40% had resided in Canada for less than three years. All participants completed validated Farsi or Arabic versions of the Generalized Anxiety Disorder 7-item scale (GAD-7), Patient Health Questionnaire 9-item (PHQ-9), Multidimensional Scale of Perceived Social Support (MSPSS), and Challenged Sense of Belonging Scale (CSBS) in their primary language.

**Results:**

Among participants, 25.70% reported symptoms of generalized anxiety (16.70% moderate, 9.00% severe), and 26.40% exhibited symptoms of major depression (20.80% moderate, 5.60% moderately severe). The overall sense of belonging was slightly above moderate, and one-third of participants reported moderate or low levels of perceived social support. Lower levels of perceived social support were significantly associated with higher anxiety and depression scores. Younger age, active job-seeking status, lower economic status, shorter length of residence in Canada, and limited English language proficiency were significant contributing factors.

**Conclusion:**

Rates of anxiety and depression in this population were considerably higher than those reported in the general Canadian population and comparable studies. These findings highlight the importance of addressing mental health and social well-being among immigrant populations and can inform the development of tailored, community-based support initiatives.

## 1. Introduction

Mental health, which includes emotional, psychological, and social well-being, plays a vital role in shaping overall health and quality of life [1]. Anxiety and depression, among the most prevalent mental health disorders, are leading contributors to the global disease burden, significantly affecting daily functioning [2]. Global statistics from the World Health Organization (WHO) highlight that over 280 million people worldwide were experiencing depression in 2023, and approximately 301 million individuals in 2019 reported anxiety [3]. In Canada, the 2022 Mental Health and Access to Care Survey (MHACS) found that more than five million people in Canada met the diagnostic criteria for a mood, anxiety, or substance use disorder in the past year [4]. These conditions impact not only individual and family well-being but also place a significant economic burden on healthcare systems and society [5,6].

A sense of belonging is a fundamental psychological need that significantly impacts individual well-being, mental health, and social functioning [7]. It is defined as the experience of being accepted, valued, and connected within a community or group. This construct encompasses both psychological and social dimensions, such as emotional attachment, group membership, and perceived social support [8,9]. Various factors contribute to a sense of belonging, including cultural identity, perceived discrimination, community engagement, and access to supportive social networks [10]. A strong sense of belonging is a critical foundation for psychological well-being [8], enhances resilience in the face of adversity, and improves overall quality of life [11]. A 2020 General Social Survey revealed that immigrants’ sense of belonging to Canada varies slightly by province, with the strongest sense of belonging observed among those living in Atlantic Canada and Ontario and the weakest among immigrants in British Columbia and Alberta [12].

Perceived social support is another essential factor influencing individual well-being and social integration [13]. It refers to an individual’s belief that they have access to assistance, care, and encouragement from others, specifically from family, friends, and significant others [14,15]. Social support is often categorized into key dimensions, including emotional support (source of comfort and empathy), instrumental support (provides tangible assistance like financial aid), informational support (involves advice and guidance), and appraisal support (helps individuals assess their situations and make informed decisions) [16,17]. High levels of perceived social support are associated with reduced psychological distress, enhanced coping mechanisms, and better health outcomes [13,18]. Immigrants, however, may experience reduced social support, likely because of limited cultural connections, structural inequities, and language barriers [2,19].

Immigration is a complex process involving relocation and adaptation to new environments that can have profound effects on mental health outcomes [2,6], particularly in individuals from non-English-speaking cultures who migrate to English-dominant societies [20,21]. While many immigrants demonstrate resilience, they also encounter unique stressors that may heighten their vulnerability to mental health issues. Acculturative stress, arising from adapting to new cultural and societal norms, has been identified as a key risk factor for mental health problems among immigrants [2,22]. Research suggests that immigrants and refugees often experience a decline in mental health over time, resulting from integration challenges and sociodemographic determinants such as employment, economic stability, and language barriers [2,23].

Mental health outcomes among migrant groups vary considerably. Population-based studies indicate that immigrants generally exhibit better overall health than both their home and host populations, with first-generation immigrants in Canada demonstrating superior physical and mental health outcomes compared to the general population [2,23]. This phenomenon is often attributed to the selective immigration process [24]. However, the findings of these studies are inconsistent [25], and research indicates that the healthy immigrant effect may not apply to mental health if the role of immigrant generation and racial and ethnic background is considered [26]. Studies also demonstrate that refugees face significantly higher risks for mental health conditions, including elevated rates of depression and anxiety, primarily due to their exposure to war, violence, forced displacement, and the uncertainty of asylum status [27–29]. Regardless of experiencing considerable psychological distress, both immigrants and refugees are less likely than their non-immigrant counterparts to seek mental health services [30–34].

Furthermore, the delivery of health services is influenced by changes in immigration patterns. In Canada, while early immigration primarily involved European settlers, a marked shift has occurred since the 1960s, with increasing migration from Asia, Africa, and Central and South America [35]. This shift in immigration trends, along with growing cultural and linguistic diversity, can significantly impact the delivery of health services, posing challenges that the healthcare system may not be adequately prepared for.

This research focused on immigrants from non-English-speaking backgrounds residing in Halifax, Nova Scotia (NS), Canada, specifically individuals who speak Farsi or Arabic. It had two main objectives: (1) to examine mental health outcomes, including anxiety and depression, as well as perceived social support and sense of belonging among these immigrants, and (2) to identify key factors contributing to the levels of these mental and psychological constructs within this population. In recent years, Canada has experienced an increase in the number of Farsi- and Arabic-speaking immigrants and refugees, particularly in the wake of multiple crises in Middle Eastern countries over the past decade. Although immigrant mental health and social well-being have been broadly studied in Canada, few studies have focused on these specific linguistic and cultural groups, whose unique experiences may place them at heightened risk for mental health issues. We hypothesized that immigrants from non-English-speaking backgrounds experience significant levels of anxiety and depression, potentially influenced by perceived social support and various sociodemographic factors.

## 2. Materials and methods

### 2.1. Participants

The research population comprised 144 participants (mean age = 36.50, SD = 11.30 years), with an equal number of men (n = 72, mean age = 37.50, SD = 12.30 years) and women (mean age = 35.50, SD = 10.10 years). Half of the participants identified Farsi as their first language, while the other half identified Arabic as their first language. Of the total sample, 74 (51.40%) were newcomers to Canada, having resided in the country for less than three years (mean = 1.80, SD = 0.90 years), whereas 70 (48.60%) had lived in Canada for four years or more (mean = 8.30, SD = 4.70 years). Regarding employment status, approximately 55.0% (n = 79; 45 men, 34 women) were employed, 22.0% (n = 32; 10 men, 22 women) were unemployed, and 23.0% (n = 33; 17 men, 16 women) were actively seeking employment. Table 1 provides additional details on the demographic characteristics of the study population, including education and English language level.

**Table 1:** Demographic characteristics of participants.

Demographic characteristics	N (%)
Gender	
Men	72 (50.0)
Women	72 (50.0)
Length of Residence in Canada	
< 3 years (newcomers)	74 (51.40)
≥ 3 years	70 (48.60)
Employment Status	
Employed	79 (54.70)
Unemployed	32 (22.20)
Actively seeking employment	33 (22.90)
Economic Status	
High (≥ 7/10)	28 (19.40)
Average (4–6/10)	91 (63.20)
Low (<4/10)	25 (17.40)
Education	
High school diploma	26 (18.10)
Undergraduate degree	60 (41.70)
Graduate degree	58 (40.30)
English language level	
High (≥8/10)	64 (44.40)
Average (5–7/10)	61 (42.40)
Low (<5/10)	19 (13.20)

Participants were recruited through social media advertisements, cultural and social organizations, and word of mouth within the Halifax Regional Municipality, NS, Canada. Data were collected at Dalhousie University and various community and cultural centers. Of the 150 individuals who took part in the study, six were excluded due to incomplete surveys. The research was approved by the Nova Scotia Health Research Ethics Board (Ethics No. 1030582), and written informed consent was obtained from all participants.

### 2.2. Procedure

Each participant took part in a single data collection session. Following the completion of sociodemographic information, participants were administered four validated self-assessment questionnaires: the Generalized Anxiety Disorder 7-item scale (GAD-7) [36–38], the Patient Health Questionnaire 9-item (PHQ-9) [12,36,39], the Multidimensional Scale of Perceived Social Support (MSPSS) [15,40,41], and the Challenged Sense of Belonging Scale (CSBS) [42]. In addition, participants rated their overall English language proficiency and economic status using a Visual Analog Scale (VAS), with scores ranging from 1 (lowest) to 10 (highest) [43,44].

#### 2.2.1. Self-assessment questionnaires.

**GAD-7.** The GAD-7 is a brief self-report scale used to screen for probable generalized anxiety disorder, with reported sensitivity and specificity of 89% and 82%, respectively [45]. It assesses the frequency of seven anxiety symptoms experienced over the past two weeks [38]. Items are rated on a scale from 0 (not at all) to 3 (nearly every day), yielding a total score ranging from 0 to 21. Scores are classified as minimal (0–4), mild (5–9), moderate (10–14), and severe anxiety (15–21) [38]. A GAD-7 score of 10 or higher, out of a total possible score of 21, is widely recognized as the threshold for identifying individuals with generalized anxiety disorder [38,46]. Validated Farsi [37] and Arabic [36] versions of the GAD-7 were used in this research.

**PHQ-9.** The PHQ-9 is a self-administered scale used to assess the severity of depression. Participants rate the frequency of nine depressive symptoms experienced over the past two weeks on a scale from 0 to 3, corresponding to responses of not at all, several days, more than half the days, and nearly every day, respectively. The total score ranges from 0 to 27 [45]. The construct and criterion validity of the PHQ-9 have been established in large sample populations, with scores inversely related to functionality. PHQ-9 scores are categorized as minimal (0–4), mild (5–9), moderate (10–14), moderately severe (15–19), and severe depression (20–27) [45,47]. A score of 10 or above on the PHQ-9 is commonly used as a clinical cutoff for detecting major depression [48]. Validated Farsi [12] and Arabic [36] versions of the PHQ-9 were used in this research.

**CSBS**. The CSBS is a validated self-report tool available in English, Farsi, and Arabic, designed to assess an individual’s emotional attachment to their environment, independent of national or cultural identity. This short scale, including four items, evaluates four distinct components: “connection (I am troubled by a feeling I have no place in this world), participation (I don’t feel that I participate with anyone or any group), identification (I feel torn between worlds), and congruence (I feel disconnected from those around me)”. Items are rated on a scale from 1 (strongly agree) to 5 (strongly disagree), where a score of 5 indicates the greatest challenge to an individual’s sense of belonging, and a score of 1 represents the least challenge. The maximum possible average score on the CSBS is 5 [42].

**MSPSS.** The MSPSS is a validated 12-item self-report questionnaire designed to assess perceived social support from three sources: family, friends, and a significant other [49,50]. Items are rated on a 7-point Likert scale, ranging from 1 (very strongly disagree) to 7 (very strongly agree), with higher scores indicating greater perceived support. The total score ranges from 12 to 84, with scores classified into three categories: low (12–35), moderate (36–60), and high (61–84) levels of perceived support [50]. This research used the validated Farsi [40] and Arabic [41] versions of the MSPSS.

### 2.3. Statistical analysis

R statistical software (version 4.3.3) was used for data preparation and analysis. The *psych* package [51] was employed to extract descriptive statistics and perform analyses, while the *ggplot2* package [52] was utilized for data visualization. Descriptive statistics were used to summarize sample characteristics. All statistical analyses were conducted at a significance level of 0.05, with p < 0.05 considered statistically significant.

Given the non-normal distribution of the data, confirmed by the Shapiro–Wilk test and potentially influenced by the small sample size, nonparametric methods were applied. The normality assessment included scores from four self-assessment questionnaires across variables such as age (young adults <40 years, older adults ≥40 years), gender (men, women), length of residence in Canada (<3 years, ≥ 3 years), education (high school diploma, undergraduate degree, graduate degree), employment (employed, unemployed, actively seeking employment), economic status (low < 4, average 5–7, high ≥8 out of 10), English proficiency (low < 5, average 5–7, high ≥8 out of 10), and native language (Farsi, Arabic).

Wilcoxon’s rank-sum tests were used to compare GAD-7 and PHQ-9 scores across gender, age groups, and ethnicity. Stepwise regression analyses were performed to identify the strongest predictors of GAD-7 and PHQ-9 scores from a set of predictors including age, gender, native language, Canadian residency duration, education level, employment status, economic status, and English language proficiency. This data-driven approach was chosen to complement theory-based reasoning by empirically identifying the most influential predictors while minimizing model complexity and the risk of overfitting, thereby enhancing interpretability and robustness. Model selection was guided by the Akaike Information Criterion (AIC) [53], a statistical measure used to evaluate how well a model fits the observed data and to select the most balanced model by weighing goodness of fit against model complexity. The process began with a base model and iteratively added or removed predictors to achieve the lowest possible AIC value.

In the reported tables, W, H, δ, and η² refer to Wilcoxon’s rank-sum statistic, Kruskal–Wallis statistic, Cliff’s Delta, and eta-squared effect size (small = 0.2, medium = 0.5, large = 0.8 out of 1.0) [54], respectively. Spearman’s two-tailed correlation coefficient (*r*) was also used to assess correlations between the questionnaire scores.

## 3. Results

**Fig 1:**
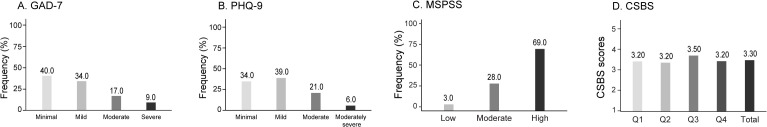
Descriptive results from the four questionnaires used in this study. **(A)** Percentage of participants reporting minimal to severe levels of anxiety (GAD-7). **(B)** Percentage of participants reporting minimal to moderately severe levels of depression (PHQ-9). **(C)** Percentage of participants reporting low (12–35), moderate (36–60), and high (61–84) levels of perceived social support (MSPSS). **(D)** Average item-based and total scores on the Challenged Sense of Belonging Scale (CSBS). Abbreviations: CSBS, Challenged Sense of Belonging Scale; GAD-7, Generalized Anxiety Disorder 7-item scale; PHQ-9, Patient Health Questionnaire 9-item scale; MSPSS, Multidimensional Scale of Perceived Social Support.

Table 2 presents the descriptive statistics for the four validated questionnaires used in this research, including the subscales and total scores. Fig 1 provides a graphical representation of descriptive results. Fig 1A illustrates the relative frequency of the participants presenting different anxiety levels, as reported in the GAD-7 questionnaire. Of the 144 participants, 40.30%, 34.0%, 16.70%, and 9.0% reported minimal, mild, moderate, and severe levels of anxiety, respectively; 25.70% exceeded the cutoff for identifying GAD [38,46]. Fig 1B depicts the proportion of participants reporting different levels of depression, reported in their PHQ-9 score. Of the total participants, 34.70%, 38.90%, 20.80%, and 5.60% reported minimal, mild, moderate, and moderately severe levels of depression, respectively. A total of 26.40% had scores surpassing the clinical threshold for detecting major depression [48]. Fig 1C demonstrates the CSBS total score (3.30 out of 5) along with the average score per question. Fig 1D depicts the percentage of participants reporting low, moderate, and high levels of perceived social support on the MSPSS, with 30.50% reporting moderate or low perceived social support. Using a VAS on a scale of 1–10, the mean economic status was 5.03 (SD = 1.70), and the mean English language proficiency level was 6.90 (SD = 2.10).

**Table 2:** Descriptive statistics for anxiety and depression scores (n = 144).

Scale	Level	Mean	SD	2SE	Range
**GAD-7**	Minimal (n = 58, 40.30%)	1.20	1.50	0.20	0–4
	Mild (n = 49, 34.0%)	7.00	1.50	0.20	5–9
	Moderate (n = 24, 16.70%)	11.20	1.40	0.30	10–14
	Severe (n = 13, 9.0%)	17.30	1.80	0.50	15–21
	Total (max = 21)	6.60	5.00	0.40	0–21
**PHQ-9**	Minimal (n = 50, 34.70%)	2.10	1.40	0.20	0–4
	Mild (n = 56, 38.90%)	6.80	1.40	0.20	5–9
	Moderate (n = 30, 20.80%)	11.80	1.30	0.20	10–14
	Moderately severe (n = 8, 5.50%)	16.60	1.50	0.50	15–19
	Total (max = 27)	6.80	4.50	0.40	0–19
**CSBS**	Connection	3.10	1.50	0.10	1–5
	Participation	3.20	1.40	0.10	1–5
	Identification	3.50	1.40	0.10	1–5
	Congruence	3.20	1.30	0.10	1–5
	Total (max = 5)	3.30	1.20	0.10	1–5
**MSPSS**	Family	23.00	5.10	0.40	4–28
	Friends	19.70	6.60	0.50	4–28
	Significant other	23.10	5.40	0.40	5–28
	Total (max = 84)	65.90	13.90	1.20	28–84
	Low (n = 4, 2.80%)	30.70	2.70	1.40	28–34
	Moderate (n = 40, 27.80%)	50.60	6.70	1.00	36–60
	High (n = 100, 69.40%)	73.40	7.80	0.80	61–84

CSBS, Challenged Sense of Belonging Scale; GAD-7, General Anxiety Disorder 7-item; MSPSS, Multidimensional Scale of Perceived Social Support; n, number of participants; PHQ-9, Patient Health Questionnaire 9-item; SD, standard deviation; SE, standard error.

### 3.1. Group comparisons

#### 3.1.1. Impact of employment status on PHQ-9 and CSBS.

**Fig 2:**
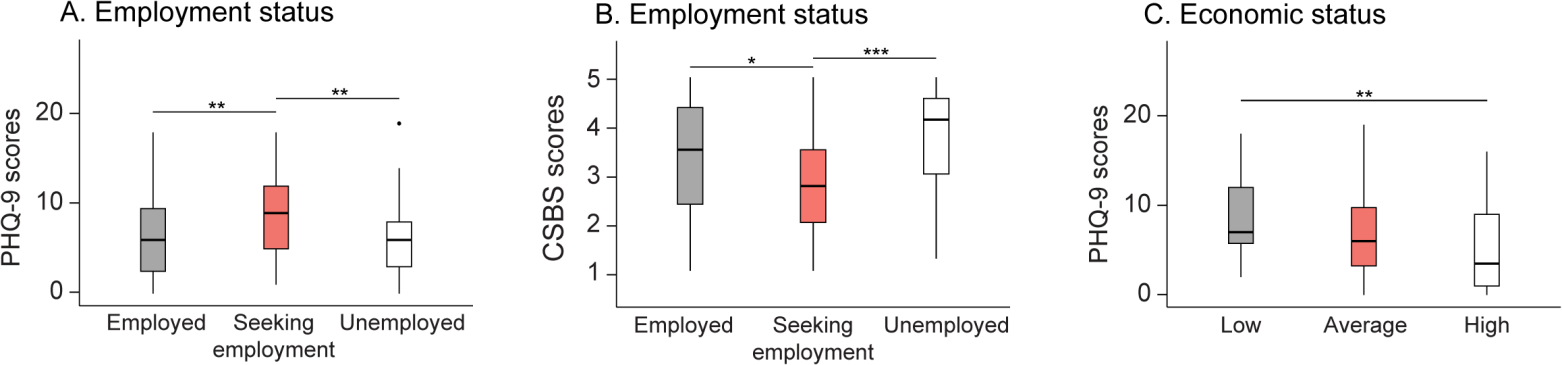
Impact of economic and employment status on PHQ-9 and CSBS scores. **(A)** Participants who were seeking employment showed higher depression scores (PHQ-9). **(B)** Job-seeking participants reported a lower sense of belonging (CSBS). **(C)** Participants with lower self-rated economic status exhibited higher depression scores (PHQ-9). Box plots display medians and interquartile ranges. Abbreviations: CSBS, Challenged Sense of Belonging Scale; GAD-7, Generalized Anxiety Disorder 7-item scale; PHQ-9, Patient Health Questionnaire 9-item scale. Asterisks indicate statistical significance: *p < 0.05, **p < 0.01, ***p < 0.001.

A significant difference was found in PHQ-9 scores among participants with different employment status (H = 9.40, *p* = 0.009, η = 0.05, Fig 2A). Pairwise comparisons indicated that the group seeking employment scored higher than both employed (W = 870.50, *p* = 0.006, δ = 0.33) and unemployed (W = 733.50, *p* = 0.007, δ = 0.40) groups. No significant difference was found between the employed and the unemployed groups (*p* = 0.751).

Similar results were found with CSBS scale, in which the seeking employment group showed a significantly lower score in total (H = 11.90, *p* = 0.003, η^2^ = 0.07; Fig 2B) and all four subscales: Connection (H = 6.30, *p* = 0.043, η^2^ = 0.03), Participation (H = 11.01, *p* = 0.004, η^2^ = 0.06), Identification (H = 9.01, *p* = 0.011, η^2^ = 0.05), and Congruence (H = 10.77, *p* = 0.005, η^2^ = 0.06). No significant difference was found between the three groups in GAD (*p* = 0.108) and MSPSS (*p* ≥ 0.299) scores.

#### 3.1.2. Impact of economic status on PHQ-9.

A significant difference was found in PHQ-9 scores among participants with different economic status (H = 7.47, *p* = 0.024, η^2^ = 0.04, Fig 2C). Post-hoc tests revealed a significantly higher PHQ-9 score in those with low (<6 out of 10) compared to high (≥7 out of 10) economic status (W = 450, *p* = 0.007, δ = 0.44). No significant difference was found in PHQ-9 scores between participants with average (6 or 7 out of 10) and high (p = 0.065) or average and low economic status (*p* = 0.111). There was no difference among participants with different economic status in GAD-7 scores (*p* = 0.444).

#### 3.1.3. Duration of residing in Canada on CSBS.

**Fig 3:**
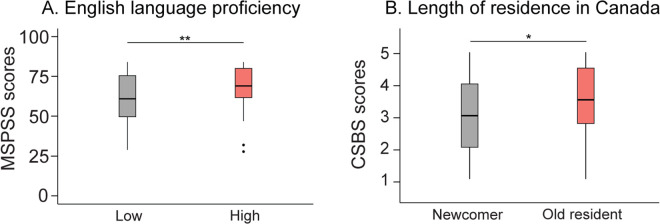
Impact of English language proficiency and length of residence in Canada on MSPSS and CSBS scores. **(A)** Participants with higher self-rated English language proficiency reported greater perceived social support (MSPSS). **(B)** Participants with a longer duration of residence in Canada reported a stronger sense of belonging (CSBS). Box plots display medians and interquartile ranges. Abbreviations: MSPSS, Multidimensional Scale of Perceived Social Support; CSBS, Challenged Sense of Belonging Scale. Asterisks indicate statistical significance: *p < 0.05, **p < 0.01.

Newcomers showed scores significantly lower than the older residents in CSBS total score (W = 2028, *p* = 0.024, δ = −0.22; Fig 3A) and its Participation (W = 1946, *p* = 0.009, δ = −0.25) and Identification (W = 1991.5, *p* = 0.014, δ = −0.23) subscales. No significant difference was found between newcomers and older residents in other questionnaires (*p* ≥ 0.265).

#### 3.1.4. English language proficiency on MSPSS.

The MSPSS total score (W = 1697, *p* = 0.002, δ = −0.31; Fig 3B) and the Family (W = 1960.5, *p* = 0.037, δ = −0.20) and Friends (W = 1668.5, *p* = 0.001, δ = −0.32) subscale scores were significantly lower in those with lower (< 7) compared to higher (≥ 7–10) English language proficiency scores. No significant difference was observed between these groups in other questionnaires (*p* ≥ 0.126).

#### 3.1.5. Other comparisons.

No significant differences were found in GAD-7 and PHQ-9 scores based on participants’ gender (*p* ≥ 0.385), age (*p* ≥ 0.123), and first language (*p* ≥ 0.416).

### 3.2. Stepwise regression analysis

Table 3 demonstrates the stepwise regression analysis results, summarizing the factors contributing to the outcomes of the GAD-7, PHQ-9, MSPSS, and CSBS questionnaires.

**Table 3:** Summary of the final models of the Stepwise Regression Analysis.

Instrument	Predictor	Estimate	SE	t	p
**GAD-7**					
	(Intercept)	10.50	1.50	6.80	< 0.001***
	Length of education	−0.20	0.10	−1.80	0.079
	Age	−0.10	0.04	−2.40	0.020*
	Employment Status (Seeking)	2.30	1.05	2.20	0.028*
	Employment Status (Unemployed)	−0.40	1.05	−0.40	0.686
**PHQ-9**					
	(Intercept)	12.20	1.90	6.50	< 0.001***
	Employment Status (Seeking)	2.30	0.10	2.40	0.019*
	Employment Status (Unemployed)	−1.40	0.90	−1.50	0.146
	Age	−0.10	0.03	−2.30	0.024*
	Economic Status	−0.40	0.20	−1.90	0.064
	Length of education	−0.20	0.10	−1.40	0.159
**CSBS**					
	(Intercept)	3.60	0.20	16.50	< 0.001***
	Migration from Farsi-speaking Countries	−0.10	0.20	−4.90	< 0.001***
	Length of residence in Canada	0.05	0.02	2.30	0.025*
	Employment status (Seeking)	−0.40	0.20	−1.90	0.059
	Employment status (Unemployed)	0.20	0.20	0.85	0.396
**MSPSS**					
	(Intercept)	41.60	5.80	7.20	< 0.001***
	Language proficiency	2.60	0.60	4.65	< 0.001***
	Age	0.20	0.10	2.40	0.018*
	Canada experience	−0.50	0.25	−1.20	0.050

CSBS, Challenged Sense of Belonging Scale; GAD-7, General Anxiety Disorder 7-item; *p*, p-value; PHQ-9, Patient Health Questionnaire 9-item; MSPSS, Multidimensional Scale of Perceived Social Support; SE, standard error; t, t-statistic. Asterisks indicate *p < 0.05, and ***p < 0.001.

#### 3.2.1. GAD-7.

The final regression model accounted for 8.82% of the variance in GAD-7 scores (Adjusted R² = 0.06, F_(4, 139) =_ 3.36, *p* = 0.012), with younger age (β = −0.09, *p* = 0.020) and active job seeking (β = 2.33, *p* = 0.028) showing significant impact on the GAD scores.

#### 3.2.2. PHQ-9.

The final regression model accounted for 14.05% of the variance in PHQ-9 scores (Adjusted R² = 0.11, F_(5, 138)_ = 4.51, *p* < 0.001). Within this model, younger age (β = −0.08, *p* = 0.024) and active job seeking (β = 2.31, *p* = 0.019) were significantly associated with depression scores.

#### 3.2.3. CSBS.

The final model explained 28.67% of the variance in CSBS scores (adjusted R-squared = 0.27, F_4, 139_ = 13.97, *p* < 0.001). Farsi-speaking group (β = −0.94, *p* < 0.001) and newer Canadian residents (β = −0.05, *p* = 0.025) showed a significantly lower sense of belonging than the Arabic-speaking and older residents, respectively.

#### 3.2.4. MSPSS.

The final model explained 14.16% of the variance in MSPSS (Adjusted R-squared = 0.1233, F_3, 140_ = 7.70, *p* < 0.001). Higher English language proficiency (β = 2.60, *p* < 0.001) and age (β = 0.24, *p* = 0.018) significantly contributed to higher MSPSS scores, while duration of residence in Canada had a near-significant effect (β = −0.05, *p* = 0.050).

### 3.3. Relationship among GAD-7, PHQ-9, and MSPSS scores

**Fig 4:**
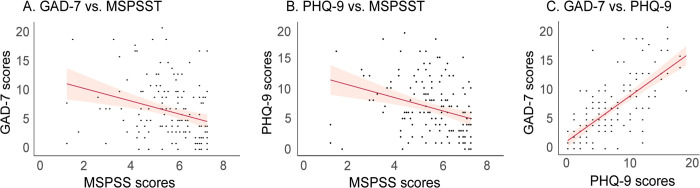
Correlations between questionnaire scores. **(A and B)** Participants with lower self-perceived social support (MSPSST) reported higher levels of anxiety (GAD-7) and depression (PHQ-9). **(C)** A strong positive correlation was observed between anxiety and depression scores. Abbreviations: GAD-7, Generalized Anxiety Disorder 7-item scale; PHQ-9, Patient Health Questionnaire 9-item scale; MSPSST, Multidimensional Scale of Perceived Social Support Total score.

Fig 4 demonstrates the correlations between MSPSS Total, GAD-7, and PHQ-9 scores. A significant negative association was found between GAD-7 and MSPSS scores (r = –0.29, *p* < 0.001; Fig 4A) and between PHQ-9 and MSPSS scores (r = –0.31, *p* < 0.001; Fig 4B). The GAD-7 and PHQ-9 total scores showed a strong positive correlation (r = 0.72, *p* < 0.001; Fig 4C).

## 4. Discussion

Four major findings emerged from this research. (1) Using GAD-7, 25.70% of participants reported moderate or severe anxiety levels that exceeded the clinical cutoff for generalized anxiety disorder (≥ 10 out of 21). (2) Based on PHQ-9, 26.40% of participants reported moderate or moderately severe levels of depression, meeting the clinical threshold for major depression (≥ 10 out of 27). Age, employment status, and economic status were identified as contributing factors to mental health outcomes. (3) Using CSBS, participants reported a moderate sense of belonging. (4) Based on MSPSS, approximately one-third of participants reported moderate or low levels of perceived social support. Employment status, duration of residence in Canada, country of origin (specifically Farsi-speaking countries), English language proficiency, and age were all factors associated with social well-being. These findings are discussed in turn.

### 4.1. Generalized anxiety and contributing factors

In this research, 25.70% of the population reported moderate (16.70%) or severe (9.0%) levels of anxiety over the past two weeks, exceeding the cutoff for GAD [38,46]. This prevalence is notably higher than that reported in the general Canadian population and comparable studies. On a general scale, 4.05% of the global population has an anxiety disorder [55]. According to the Canadian Community Health Survey (CCHS, 2022), the proportion of Canadians aged 15 years and older with generalized anxiety disorder doubled from 2.6% in 2012 to 5.2% in 2022 [4]. A systematic review of 48 studies found point prevalence rates ranging from 3.8% to 25.0%, particularly among women, young adults, and individuals with chronic illnesses [56]. In region-specific research, a German nationwide study reported a 12-month prevalence of 15.3% for anxiety [57], and a U.S. national survey documented a lifetime prevalence of 28.8% [58]. These comparisons underscore the disproportionately high burden of anxiety among our participants compared to both national and international statistics, likely influenced by migration-related stressors such as loss of social networks, underemployment, and the psychological impact of cultural transition. These findings align with previous Canadian research that has emphasized the mental health vulnerabilities of immigrants, particularly those from culturally and linguistically diverse backgrounds [2,59].

#### 4.1.1. Age.

Our findings indicate that age is a significant predictor of anxiety, with younger adults reporting higher levels of anxiety. This finding is consistent with existing literature on both immigrant and general populations in Canada and internationally. Several studies across countries have reported high levels of psychological distress and anxiety among young refugees and immigrants [2,28,60]. For example, a study in Sweden [61] observed elevated anxiety symptoms among younger adults, especially in economically disadvantaged or transitional life phases. A study in Australia also found similar age-related patterns of anxiety, particularly among individuals navigating immigration, education, or job-related uncertainties [62].

A possible explanation for this trend is that younger adults may lack well-developed coping mechanisms, emotional regulation strategies, and stable support networks that typically strengthen with age [63,64]. In immigrant contexts, these deficits are further exacerbated by disconnection from extended family, unfamiliar cultural environments, and language barriers [2,42,65]. Moreover, younger immigrants often experience more “cultural distance” and identity-related stress, which may contribute to internal conflict, lowered self-esteem, and heightened anxiety and depressive symptoms [66,67].

#### 4.1.2. Employment status.

In this research, 54.90% of participants were employed, 22.20% were unemployed, and 22.90% were actively seeking employment, indicating an employment rate slightly below the national average of 62.1% for individuals aged 15 and older [68]. Our findings reveal that active job seekers report significantly higher anxiety levels compared to their employed or non-seeking counterparts. This finding supports existing literature acknowledging that employment serves as a source of social identity, structure, and psychological stability [69]. Immigrants who actively search for employment often face additional barriers, including language proficiency challenges, non-recognition of foreign credentials, and limited professional networks. It has been shown that such individuals are at increased risk of psychological distress due to repeated job rejections, precarious job offers, and financial uncertainty [19,27,70].

### 4.2. Depression and contributing factors

In our research, 26.40% of participants reported moderate (20.80%) or moderately severe (5.60%) depressive symptoms, suggesting the presence of clinically significant major depression [48]. These findings highlight a significant mental health concern, indicating that nearly two-thirds of the sample are experiencing elevated levels of depressive symptoms. Compared to national and international data, this rate appears substantially higher, exceeding what can be attributed to methodological variations such as sample size and assessment tools. According to CCHS (2022), the proportion of Canadians aged 15 years and older reporting major depressive symptoms increased from 4.7% in 2012 to 7.6% in 2022 [4]. The WHO estimates that approximately 3.8% of the global population experiences depression, with higher rates observed among adults, particularly women [71]. A meta-analysis of studies between 1994 and 2014 estimated the aggregate point, one-year, and lifetime prevalence of depression and calculated prevalences of 12.9%, 7.2%, and 10.8%, respectively [72]. Other community-based surveys, including 8.4% in the U.S. [73], 6.0% in Germany [74], and 4.1% in Australia [75] also report considerably lower depression rates than those observed in our sample. The high rate of depressive symptoms in the present study likely stems from migration-related stressors that disproportionately affect culturally and linguistically diverse immigrants [2,76].

#### 4.2.1. Age.

A significantly higher frequency of depression in younger than older immigrants aligns with Canadian national data and international trends, showing higher vulnerability of younger adults to depression, potentially due to transitional life stressors and less developed coping strategies [1,63,64]. In immigrant populations, these vulnerabilities are further exacerbated by the stress of adapting to a new cultural and social environment, separation from family, and difficulties establishing financial and emotional independence [2,28]. Internationally, a Swedish population-based study identified a higher depression risk in young adults from migrant backgrounds, particularly refugees who experienced disrupted education and limited social support [61].

#### 4.2.2. Economic status.

Our data show the link between a lower economic status and higher levels of depression. Economic insecurity has been shown as a critical social determinant of health that contributes to chronic stress, reduced access to resources, and feelings of powerlessness, all of which can affect the onset and persistence of depression [77]. Among immigrant populations, challenges such as credential recognition, language barriers, and discrimination in the labor market may further intensify their financial strain and limit employment opportunities and income stability [19,76]. In this regard, CCSH (2020) indicates that individuals in the lowest income quintile are more than twice as likely to report poor mental health compared to those in the highest quintile [78]. A systematic review also reported that poverty and low socioeconomic status were consistently associated with higher rates of depression [79].

#### 4.2.3. Employment-seeking status.

Our research indicates that participants actively seeking employment report higher depressive symptoms than both employed and unemployed individuals. This group potentially faces distinct challenges in the ongoing job search efforts, such as persistent uncertainty, repeated rejection, and systemic barriers such as language limitations and unrecognized credentials. These stressors, particularly prevalent among immigrants, can intensify feelings of inadequacy and psychological strain [69,80,81]. where qualified individuals are unable to secure work matching their education and experience [69].

### 4.3. Sense of belonging and influencing factors

The subjective experience of deep connection with social groups, physical places, and individual and collective experiences, referred to as a sense of belonging, is a fundamental human need that underpins various mental, physical, social, economic, and behavioral outcomes [8,82]. When this sense of belonging is challenged or diminished, individuals may experience perceived social disconnection, which can act as a psychological stressor and threaten overall well-being [83,84]. Pfaff-Czarnecka (2013) conceptualizes belonging as “an emotionally charged, ever-dynamic social location,” emphasizing its fluid and often contested nature rather than treating it as a fixed attribute. Based on this framework, the CSBS scale was developed and validated in Germany across three languages (English, Arabic, and Farsi/Dari) to measure individuals’ perceptions of challenged belonging [42]. The scale includes four central components. “Connection” refers to the feeling of having a place within a social, national, or geographical context [85,86] and its absence is often associated with loneliness and insecurity [87,88]. “Participation” captures the reciprocal sense of being accepted and valued in a social system [9,42,89]. “Identification” involves the extent to which one feels personally aligned with their surroundings [82,90]. “Congruence” also refers to the harmony between one’s current place of residence and personal life story, and a coherent alignment that may become disrupted during cultural transitions [82,91].

In our study population, the total CSBS score (3.27) and its four subscale scores, Connection (3.15), Participation (3.22), Identification (3.49), and Congruence (3.21), fell slightly above the midpoint of the 5-point scale. This may indicate a moderate sense of belonging within the Canadian culture and reflect a degree of uncertainty or ambivalence. Specifically, the relatively lower scores on the Connection and Congruence subscales suggest difficulties in forming meaningful interpersonal relationships or feeling emotionally aligned with the prevailing cultural and social norms. The Participation score points to moderate engagement in social and community activities, possibly due to barriers such as language difficulties, lack of confidence, or unfamiliarity with local customs. The Identification subscale score indicates that while participants may have continued to feel connected to their original cultural identity, they have also begun to see themselves as part of the new society, even if this emerging identity is not yet fully secure. These findings indicate that many participants were in an interstitial phase of adaptation, in which a solid sense of belonging was developing but remained incomplete.

These patterns are reflected in recent Canadian national data. In a 2024 national survey, approximately 50% of the population reported a “very strong” or “somewhat strong” sense of belonging, while 35% indicated a “somewhat weak” or “very weak” sense of belonging, and 10–15% had “no opinion.” In NS specifically, the corresponding figures were 52.7%, 33.4%, and 13.9%, respectively. These results suggest that, while half of the population in Canada reports a strong sense of belonging, a considerable portion still experiences weak or uncertain connections to their communities, underscoring the importance of initiatives that promote social cohesion and inclusive belonging. Additionally, an analysis of CCHS data (2007–2014) for the Ontario province revealed the weakest sense of local community belonging in Chinese residents compared to other nations, underscoring the role of ethnicity in perceived sense of belonging and the need for ethnically tailored and culturally appropriate supports [92].

#### 4.3.1. Length of residence in Canada.

Our findings demonstrated that a shorter “duration of residence in Canada” was significantly associated with a lower sense of belonging, as reflected in both total CSBS scores and specific subscales assessing Identification and Participation. These results suggest that recent immigrants may experience greater difficulty in constructing a coherent identity and engaging meaningfully with the host society, while longer residence in the host country is positively linked with social integration and social well-being [93,94]. This is consistent with Berry’s (1997) acculturation theory, which posits that immigrants often face cultural dissonance and social detachment during the initial phases of adaptation [95]. Limited residency duration in Canada may constrain opportunities to form social networks, become familiar with institutional systems, and develop emotional attachment to local communities [96]. Early resettlement is also characterized by language barriers and cultural displacement, which further influence participation and the feeling of ambivalence between cultural identities [93,94].

#### 4.3.2. Employment status.

Our analyses showed employment status as a significant determinant of a diminished sense of belonging. Notably, participants actively seeking employment reported significantly lower CSBS total scores, as well as lower scores across all four subscales, compared to both employed individuals and those unemployed but not seeking work. This finding suggests that the experience of job-seeking, often marked by uncertainty, rejection, and systemic barriers, may intensify psychological distress and diminish social integration [94]. The challenges faced by job seekers, such as limited access to professional networks, unrecognized foreign credentials, language barriers, and potential discrimination, can contribute to a diminished sense of social value [97]. In contrast, individuals not engaged in the job market may be less exposed to these daily stressors and may rely more on informal social supports or find stability through other life roles. Beyond financial security, employment builds a pathway for social participation, identity construction, and institutional engagement [98]. Our results support Hynie’s (2018) assertion that “employment is a cornerstone of immigrant integration” [99] and Beiser and Hou’s (2001) findings that early employment is predictive of long-term social outcomes [81].

#### 4.3.3. Ethnicity (Farsi-speaking vs. Arabic-speaking Immigrants).

Ethnic background also influenced participants’ sense of belonging. Farsi-speaking immigrants reported significantly lower scores on the CSBS overall and across all four subscales compared to Arabic-speaking peers. Several factors may explain this disparity. The larger and more established Arabic-speaking community in Halifax likely offers stronger ethnic support networks and plays a key role in fostering emotional security and facilitating integration. In contrast, Farsi-speaking immigrants, primarily from Iran and Afghanistan, represent smaller and newer communities whose experiences of economic or political trauma, war, and forced displacement may further disrupt social cohesion and identity formation [100]. This is consistent with findings from Noh and Kaspar (2003), who reported that the size and cohesiveness of ethnic communities significantly influence immigrants’ social integration [101]. Furthermore, sociopolitical tensions and experiences of racialization may result in internalized marginalization for Farsi speakers, compounding their challenges in establishing a sense of belonging.

### 4.4. Perceived social support and influencing factors

In our study, the majority of participants (approximately 70%) reported high levels of perceived social support, while 28% reported moderate support, and only a small proportion (around 3%) indicated low support. Notably, participants reported higher scores on the “Family” and “Significant Other” subscales of MSPSS, while the “Friends” subscale received comparatively lower ratings. This distribution suggests that, within this population, familial and intimate relationships serve as the primary sources of emotional and instrumental support, which may be observed in many immigrant groups from countries in the Middle East, such as Syria, Iran, Afghanistan [102–104]. A lower score in the Friends subscale may indicate barriers to building broader social connections within Canadian society, possibly due to language limitations or limited opportunities for social integration.

Self-perceived social support plays a central role in psychological resilience, stress regulation, and overall well-being, especially among immigrant populations adapting to new sociocultural contexts [14]. According to Canada’s 2024 national surveys, immigrants admitted in 2005 or later are more likely to report financial hardship, with 43% indicating difficulty meeting basic needs compared to 29% of more established immigrants and non-immigrants. In this survey, economic uncertainty was especially pronounced among racialized groups, including West Asian (48%), South Asian (43%), Latin American (42%), Black (40%), Arab (38%), and Filipino (35%) Canadians, potentially influenced by the availability of social support [105].

#### 4.4.1. Language proficiency.

In the context of immigrants, access to community services and social networks often hinges on effective communication. In our study, individuals with lower English language proficiency reported significantly lower total scores on the MSPSS, particularly in the Friends subscale, followed by the Family subscale. This suggests that limited language skills may directly influence the knowledge and perception of available social support. While family support may remain accessible due to shared linguistic and cultural backgrounds, forming and maintaining friendships in a new linguistic environment appears to be more challenging [106].

#### 4.4.2. Age.

Age emerged as an impactful factor, with younger adults reporting significantly lower MSPSS scores than older individuals. This pattern may be attributed to several age-related differences in coping strategies, emotional regulation, and the maturity of social networks [107]. Younger immigrants may be particularly susceptible to perceived isolation during the early stages of acculturation, especially if they are navigating school, employment, or identity challenges without strong support systems in place [95]. In contrast, older adults may benefit from more established familial roles or longer exposure to adaptive coping resources, enabling them to sustain or rebuild social networks post-migration [108]. These findings support the evidence suggesting that perceived social support varies across age groups [109] and should be addressed through age-sensitive support programs.

In our research, lower levels of perceived social support, as measured by the MSPSS, were associated with higher anxiety and depression scores. A strong positive correlation between anxiety and depression was also observed. These findings are consistent with existing literature that emphasizes the critical role of social support in mental health, particularly among immigrant populations [110,111]. Perceived social support acts as a protective factor by offering emotional, informational, and practical resources that help buffer individuals against stress. For immigrants, limited social support can intensify feelings of isolation and stress, increasing their vulnerability to anxiety and depression [112,113]. The strong correlation between anxiety and depression further supports previous research showing that these conditions frequently co-occur and often stem from shared underlying factors [2], as reflected in the present research.

### 4.5. Significance, strengths, limitations, and directions for future research

#### Significance.

The findings draw attention to measurable disparities in the mental health and social well-being of immigrants affected by socioeconomic factors. These results are especially relevant in the context of changes in Canadian immigration patterns over recent decades, where increasing numbers of immigrants are arriving from Asia and the Middle East. The elevated rates of anxiety and depression, particularly among younger adults, those seeking employment, and those with lower economic status, raise important questions about systemic barriers related to labor market access and social support. This is particularly relevant in provinces such as NS, where Arabic has become the third most commonly spoken language after English and French [114,115] and where a significant number of refugees and immigrants from countries such as Iran and Afghanistan have resettled in recent years.

#### Strengths.

Our use of validated questionnaires in the participants’ first language, combined with the application of multivariable statistical modeling, enhances the depth and reliability of the findings. Furthermore, unlike studies that generalize immigrant populations, this research focuses specifically on non-English-speaking subgroups, offering targeted data that can help inform efforts to address the employment and integration challenges faced by these populations.

#### Limitations.

Despite its contributions, this research has several limitations that must be acknowledged. The cross-sectional design of our research restricts the ability to infer causality between sociodemographic variables and mental health outcomes. A non-immigrant comparison group and a mixed methods approach also could enhance the depth and richness of the findings. Additionally, focusing on a single city may limit the generalizability of findings across Canada.

### 4.6. Directions for future research

Building on these limitations, future research would benefit from larger, longitudinal, and multisite studies to track the mental health and social well-being of immigrants over time. Such an approach would offer deeper insights into how these factors interact and influence long-term integration outcomes. In addition, mixed-methods approaches could provide deeper insights into personal experiences, cultural beliefs, and community-level factors that quantitative tools might overlook.

## 5. Conclusions

This research examined the state of mental health and social well-being, along with contributing sociodemographic factors, among adult Farsi- and Arabic-speaking immigrants in Canada. The findings indicated that approximately one-fourth of participants exhibited signs of generalized anxiety disorder and symptoms of major depression. Participants generally reported a slightly above moderate sense of belonging, and approximately one-third of the sample reported moderate or low levels of perceived social support. Key sociodemographic factors influencing these outcomes included younger age, being actively engaged in job seeking, low economic status, shorter duration of residence in Canada, migration from Farsi-speaking countries, and limited English-language proficiency. These findings highlight the complex interplay of individual and contextual factors affecting immigrant mental health, underscoring the need for future longitudinal and mixed-methods research to guide the development of culturally sensitive and tailored interventions.
